# Digital Workflow for Immediate Implant Placement and Chairside Provisionalization in the Esthetic Zone

**DOI:** 10.1155/2022/5114332

**Published:** 2022-04-01

**Authors:** Vincent J. J. Donker, Gerry M. Raghoebar, Arjan Vissink, Henny J. A. Meijer

**Affiliations:** ^1^University of Groningen, University Medical Center Groningen, Department of Oral and Maxillofacial Surgery, Groningen, Netherlands; ^2^University of Groningen, University Medical Center Groningen, Department of Implant Dentistry, Groningen, Netherlands

## Abstract

**Introduction:**

Immediate implant placement and immediate chairside provisionalization in the esthetic zone require meticulous treatment planning. A digital workflow that combines intraoral scans and a cone beam computed tomography scan can be used to visualize the surgical and restorative aspects of the treatment and to plan a prosthetically driven implant position. A digital workflow in implant dentistry enables the prefabrication of an individualized CAD/CAM temporary restoration, based on the planned implant position. This could be a predictable method to deliver a screw-retained temporary restoration, directly after static computer-assisted immediate implant surgery. *Interventions*. Three patients with a failing tooth in the maxillary esthetic zone were treated with immediate implant placement and chairside provisionalization using this digital workflow. After 3 months, a final restoration was placed. Clinical, radiographic, and patient-reported outcome measures were collected prior to implant treatment, 6 weeks after placing the temporary restoration and then 1 month and 1 year after placing the final restoration. *Outcomes*. At the 1-year follow-up, healthy soft tissues were observed, and peri-implant bone levels were stable. Patient satisfaction after the treatment was high.

**Conclusion:**

The three reported cases demonstrate the potential for predictable immediate implant placement and chairside provisionalization using a digital workflow.

## 1. Introduction

When a failing tooth in the esthetic zone needs to be removed, it can be replaced with an implant-supported restoration. There is a growing tendency towards immediate implant placement after tooth extraction. This reduces treatment time because implant osseointegration and extraction socket healing occur simultaneously, while also preserving the hard and soft peri-implant tissues [1]. When immediate implant placement is combined with buccal socket grafting, a stable bone layer can be maintained buccally of the implant, regardless of the presence of a preoperative buccal bone defect [2, 3]. To achieve an optimal esthetic outcome, the peri-implant soft tissues must be conditioned during the socket healing. This requires customization of the emergence profile by contouring it with a temporary restoration [4, 5]. Good clinical outcomes can be achieved with immediate implant placement and immediate provisionalization, resulting in high patient satisfaction [6, 7].

However, immediate implant placement and immediate provisionalization require meticulous treatment planning. To assist the clinician with this, a digital workflow can be used [8]. Such a digital workflow combines the information of the patients' hard and soft tissues by integrating intraoral scans and a cone beam computed tomography (CBCT) scan. This allows the clinician to visualize the surgical and restorative aspects of the treatment and to plan a prosthetically driven implant position. Consequently, an individualized temporary restoration can be designed and prefabricated. Using static computer-assisted implant surgery with a surgical template, the implant can then be placed in the correct three-dimensional position and the individualized screw-retained temporary restoration can be delivered. The whole digital workflow could be a predictable method for immediate implant placement and chairside provisionalization.

The objective of this case report is to describe a treatment with this digital workflow for immediate implant placement and chairside provisionalization in the esthetic zone and to report the clinical, radiographic, and patient-reported outcome measures of three cases with a 1-year follow-up after placing the final restoration.

## 2. Study Design

Recruitment of patients, treatment, and follow-up took place in the Department of Oral and Maxillofacial Surgery at the University Medical Center Groningen (UMCG), the Netherlands, between August 2020 and December 2021. The research protocol was approved by the Medical Ethics Review Board of the UMCG (METc 2020/014). This manuscript was written following the CARE guidelines for case reports [9].

## 3. Patient Information and Clinical Findings

Patients were eligible for inclusion if they met the following criteria: at least 18 years of age when being treated, in need of a single tooth implant-supported restoration in a postextraction site in the anterior region of the maxilla, the bone height of the planned implant site apicopalatal of the extraction socket was sufficient (≥4 mm, measured on a CBCT scan) to allow immediate implant placement, suitable anatomical conditions (mesial-distal, buccal-palatal, and interocclusal space) to place an anatomically designed screw-retained restoration, and complies with good oral hygiene practices. Patients with medical and general contraindications for the surgical procedure, smoking habits exceeding 10 units a day, severe bruxism with dysfunctional tendencies, the presence of acute untreated periodontitis in the implant site or adjacent tissue, acute infections in the planned implant site or adjacent tissue, and a history of local radiotherapy to the head and neck region were excluded.

Three consecutively recruited patients agreed to participate in the study (Table 1). An explanation of the costs, benefits, and risks of an implant-supported restoration, and possible alternative treatment options was given. Written informed consent was obtained from all the patients before being enrolled for implant treatment.

## 4. Timeline

The patients underwent immediate implant placement and chairside provisionalization according to the treatment procedures described in Table 2. One case is presented for illustrative purposes.

## 5. Diagnostic and Planning Procedures

**Figure 1:**
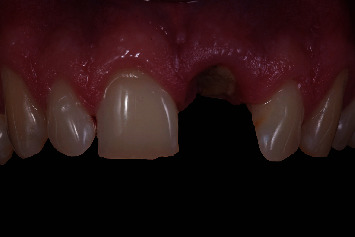
Clinical view of the patient with a root fracture of the upper left central incisor. The root is still in situ.

**Figure 2:**
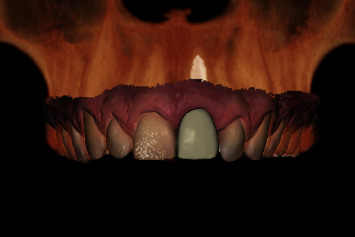
Digital set-up of the upper left central incisor, based on a superimposed intraoral scan and CBCT scan.

**Figure 3:**
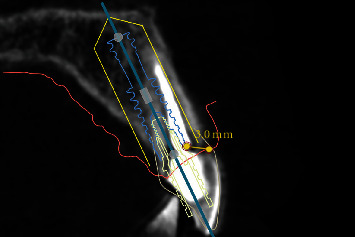
Sagittal view of the CBCT scan showing the prosthetically and biologically driven implant planning.

Prior to the implant treatment, intraoral scans (TRIOS 3, 3Shape, Copenhagen, Denmark) of the upper jaw, lower jaw, and occlusion were made, as well as intraoral photographs (Figure 1) and a CBCT scan (ProMax 3D Max ProFace, Planmeca, Helsinki, Finland). The voxel size of the CBCT scan was 0.2 mm, and the field of view was 130 × 55 mm. The intraoral scans and CBCT Digital Imaging and Communications in Medicine (DICOM) file were superimposed with an implant planning software (DTX Studio Implant, Version 3.5, Medicim, Mechelen, Belgium) to create an individualized digital set-up of the failing tooth (Figure 2). The prosthetically driven implant position was planned in a palatal position with ≥2 mm distance between the implant and the buccal crest [10] and at a depth of 3-4 mm apical of the prospective restorative zenith point [11]. The implant length was chosen based on the available bone height apicopalatal of the extraction socket (Figure 3).

**Figure 4:**
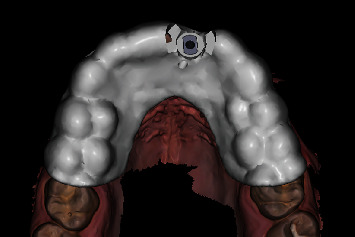
CAD surgical template based on the intraoral scan and the implant planning.

**Figure 5:**
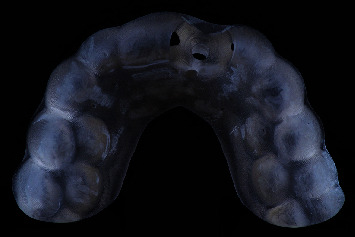
Printed surgical template.

**Figure 6:**
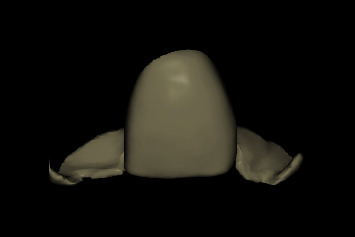
CAD temporary shell restoration with two lateral wings to aid seating.

A pilot-drill surgical template to facilitate static computer-assisted implant surgery was designed with the implant planning software (Figure 4) and manufactured by a dental laboratory (Dental LT Clear Resin, Formlabs, Somerville, MA, United States) (Figure 5). Furthermore, the dental laboratory milled a multilayered polymethyl methacrylate (PMMA) (Vipi Block Trilux, Vipi Odonto Products, Pirassununga, Brazil) temporary shell restoration based on the individualized digital set-up (Figure 6) using a dental computer-aided design (CAD) software (DTX Studio Lab, Version 1.10, Medicim).

## 6. Surgical Procedures

The patient was instructed to start oral disinfection with a 0.12% chlorhexidine mouthwash twice daily on the day before surgery and to continue for seven days. One hour prior to surgery, the patient took a prophylactic antibiotic (amoxicillin 2 g or clindamycin 600 mg in case of penicillin allergy). The surgical procedure was performed using local anesthesia (Ultracain D-S forte, Sanofi-Aventis Deutschland GmbH, Frankfurt am Main, Germany).

**Figure 7:**
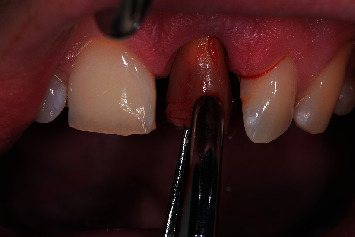
Minimally traumatic removal of the failing tooth using forceps with rotational movement.

**Figure 8:**
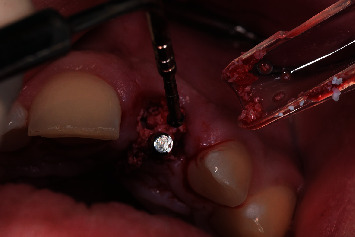
Buccal socket grafting with the last used drill placed in the implant site.

**Figure 9:**
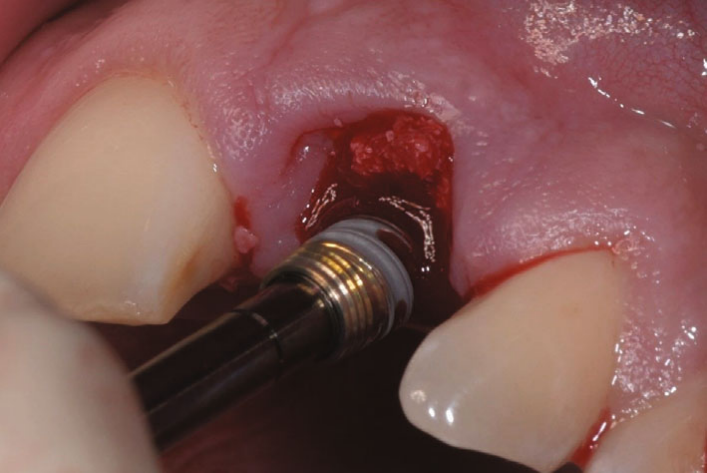
Insertion of the implant in the grafted alveolar socket.

The periodontal ligament was detached by careful intrasulcular incision. A periotome and forceps (rotational movement only) were used to remove the failing tooth as minimally traumatic as possible (Figure 7). Any periodontal ligament remnants and granulation tissue were cleaned away with a bone curette and sterile gauze pads. The implant site was prepared flapless using the surgical template. With the last drill placed in the prepared implant site to prevent congestion, the jumping gap between the buccal crest and the implant site was grafted with a 1 : 1 mixture of bovine bone (Geistlich Bio-Oss, Geistlich Pharma AG, Wolhusen, Switzerland) and autogenous bone (Figure 8). After removing the drill from the grafted alveolar socket, a tapered implant with a conical connection (NobelActive TiUltra, Nobel Biocare AB, Gothenburg, Sweden) was inserted according to the manufacturer's instructions (Figure 9). Primary implant stability was attained with an insertion torque of ≥45 Ncm, verified with a manual torque controller (NobelActive Manual Torque Wrench Surgical, Nobel Biocare AB). The surgical procedures were performed by one oral and maxillofacial surgeon (GMR).

## 7. Restorative Procedures

**Figure 10:**
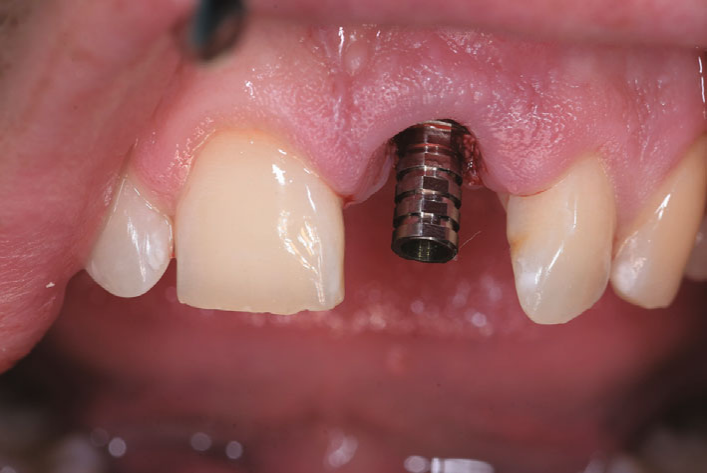
Temporary abutment snapped onto the implant.

**Figure 11:**
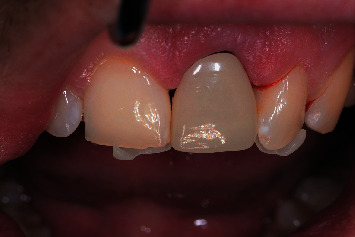
Temporary shell restoration with the lateral wings seated on the adjacent teeth.

**Figure 12:**
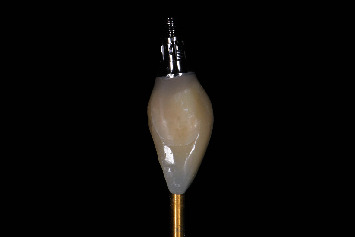
Emergence profile of the finished and polished temporary restoration.

**Figure 13:**
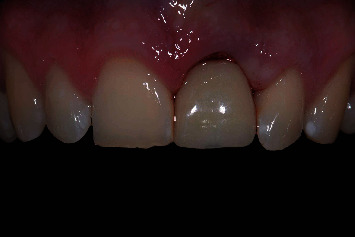
Clinical view after immediate implant placement and immediate provisionalization.

The implant was restored chairside with a temporary restoration, immediately after implant placement. A temporary abutment (Temporary Snap Abutment Engaging CC, Nobel Biocare AB) trimmed to the correct length was snapped onto the implant (Figure 10). The temporary shell restoration was placed in position with the lateral wings seated on the adjacent teeth (Figure 11) and was connected to the temporary abutment with a dentine shaded composite resin (Filtek Supreme XTE, 3 M, Saint Paul, MN, United States). After light curing, the temporary restoration was removed with the abutment from the implant. The lateral wings were trimmed, and a screw-access hole was prepared in the restoration (Apical Drill, Nobel Biocare AB). The cervical part of the restoration was contoured with composite resin to create an emergence profile to support the adjacent papillae [12] (Figure 12). The restoration was polished using polishing rubbers (Diacomp Plus Twist, EVE Ernst Vetter GmbH, Keltern, Germany) and tightened on the implant with a torque value of 35 Ncm using a manual torque controller (Manual Torque Wrench Prosthetic, Nobel Biocare AB). The screw access hole was sealed with polytetrafluoroethylene (PTFE) tape and composite resin (Figure 13). The temporary restoration was free from centric and eccentric movements and the patient was instructed to avoid excess force during the healing period. Oral hygiene instructions were given.

**Figure 14:**
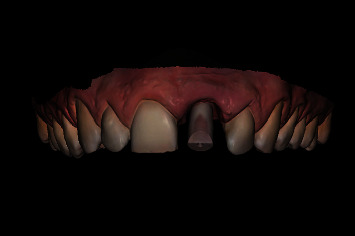
Intraoral scan with a scanbody screwed onto the implant.

**Figure 15:**
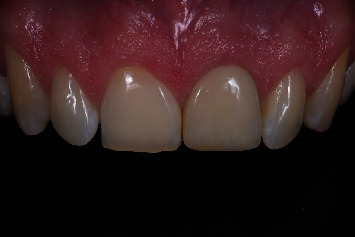
Final upper left central incisor restoration after 1 year in function.

After 3 months, an intraoral scan was made directly after removing the temporary restoration to capture the emergence profile. Furthermore, an intraoral scan was made with a scanbody (Elos Accurate Scan Body, Elos Medtech, Gothenburg, Sweden) after screwing it onto the implant (Figure 14). The dental laboratory designed a final restoration using a dental CAD software (DTX Studio Lab). The restoration was screw-retained with a CAD/CAM angulated screw-channel zirconia abutment, mechanically attached to a titanium base (NobelProcera ASC Abutment, Nobel Biocare AB), and buccally veneered with porcelain. The final restoration was tightened onto the implant with a torque value of 35 Ncm (Figure 15). The screw access hole was sealed with PTFE tape and composite resin. After placing the final restoration, oral hygiene instructions were given. All the laboratory procedures were carried out by one dental laboratory. The restorative procedures were performed by two dentists (HJAM, VJJD).

## 8. Follow-Up and Case Outcomes

Clinical, radiographic, and patient-reported outcome measures were collected prior to the treatment (Tpre), 6 weeks after placing the temporary restoration (Ttemp), and then 1 month (T1) and 1 year (T12) after placing the final restoration. No implants or restorations had been lost, and no technical or biologic complications had occurred at T12 resulting in a survival rate and success rate of 100% for the implants and the restorations according to the modified United States Public Health Service criteria for evaluating implant-supported restorations [13].

**Figure 16:**
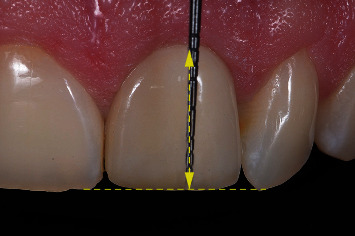
MBML measurement on a calibrated intraoral photograph with reference lines.

The soft tissue outcomes were assessed at T12 with the modified Plaque Index [14], the modified Sulcus Bleeding Index [14], the Gingival Index [15], keratinized mucosa width (KMW) measured to the nearest 1 mm with a periodontal probe [16], the Papilla Index [17], and the pocket probing depth at four sites (mesial, distal, buccal, and palatal) measured to the nearest mm (the highest value is reported). Midbuccal mucosal level (MBML) changes were assessed at Tpre and T12 on intraoral photographs (Nikon D750, Nikon Corporation, Tokyo, Japan) taken with a periodontal probe held close to and parallel to the tooth for calibration purposes (Figure 16). MBML changes were measured with a raster graphics editing software (Adobe Photoshop, Version 23.0, Adobe Inc. San Jose, CA, Unites States), and were defined as a change in the vertical distance in mm measured from the zenith point to a reference line drawn between the incisal edges of the two adjacent teeth [7]. The esthetic outcome at Tpre, Ttemp, and T12 was assessed with the modified Pink Esthetic and White Esthetic Score (PES/WES) [18] from intraoral photographs with a contrastor (Flexipalette, Smile Line SA, Saint-Imier, Switzerland). The WES at Tpre was not assessed for one case due to the absence of a clinical crown.

**Figure 17:**
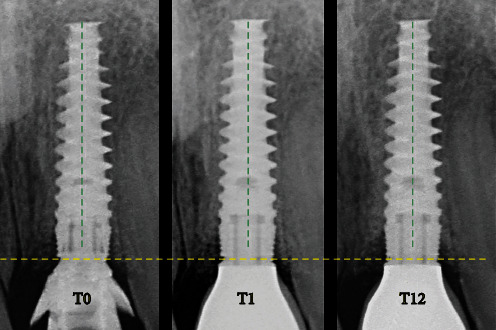
MBL measurement on calibrated intraoral radiographs at T0, T1, and T12.

**Figure 18:**
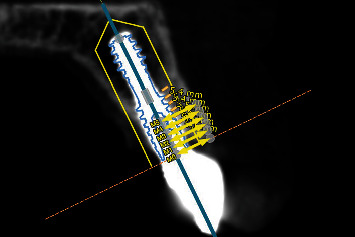
BBT measurement at the upper 5 mm of the implant on a CBCT scan at T12.

Marginal bone level (MBL) changes from T1 to T12 were assessed on calibrated intraoral radiographs with a long cone paralleling technique. The MBL change was measured by a dedicated software (DicomWorks, Biomedical Engineering, UMCG, The Netherlands), and was defined as a change in the vertical distance in mm measured from the implant shoulder to the first bone to implant contact on the mesial and distal site of the implant [6] (Figure 17). Buccal bone thickness (BBT) at T12 was assessed on a CBCT scan. The exact implant position in the CBCT DICOM file was determined with multimodality image registration using information theory (MIRIT) in a medical imaging software (Maxilim, Version 2.3, Medicim). The BBT was measured by an implant planning software with a dedicated research add-on for the measurements (NobelClinician, version 2.1, Nobel Biocare Belgium NV, Mechelen, Belgium) and was defined as the horizontal distance in mm measured from the implant outline to the buccal bone outline. The area of interest was the upper 5 mm of the implant, starting at the implant neck (Figure 18). This validated BBT measurement method was described in detail by Slagter et al. [19].

The patient-reported outcome at Tpre, Ttemp, and T12 was assessed with a questionnaire on overall satisfaction based on a 10-point rating scale. All the data were collected by one observer (VJJD). The mean values and standard deviations were calculated using statistical software (IBM SPSS Statistics, Version 23.0. Armonk, NY, United States). The outcome measures of all cases are shown in Table 3.

## 9. Discussion

This case report described a treatment with a digital workflow for immediate implant placement and chairside provisionalization in three patients with a failing tooth in the maxillary esthetic zone. The clinical, radiographic, and patient-reported outcome measures up to 1 year after placing the final restoration were favorable, which is similar to the 1-year results of immediate implant placement and provisionalization studies with a conventional workflow, conducted in the same center [6, 7]. However, the digital workflow used in this case report offered several advantages over a conventional treatment.

In the diagnostic and planning phase, the superimposed intraoral scans and CBCT scan yielded simultaneous visualization of the hard and soft tissues with a digital set-up of the failing tooth. This facilitated the planning of a prosthetically driven implant position and, at the same time, a priori assessment of the available bone height at the implant site, apicopalatal of the extraction socket. It is suggested that at least 4 mm of bone is needed to attain primary implant stability during immediate implant placement [20]. Hence the inclusion criterion for the present case report. Primary stability of the implant was indeed reached in all three cases, which allowed immediate provisionalization. The digital planning also aided in selecting an implant diameter and length that fit within the biologic boundaries. The planned distance of ≥2 mm between the implant and the buccal crest and the subsequent buccal socket grafting resulted in a mean BBT ranging from 2.35 ± 1.02 mm to 2.88 ± 0.66 mm at the 1-year follow-up. These findings differ from another study that evaluated the BBT 1 year after immediate implant placement and provisionalization combined with buccal socket grafting [2]. The mean BBT in the present cases was 0.62 mm to 0.92 mm thicker in the upper 5 mm along the axis of the implant. In the previously mentioned study, the implant planning and ensuing pilot-drill surgical template fabrication was based on a stone cast with a conventional wax set-up. Possibly, the combined visualization of the hard and soft tissues in the digital workflow used in this case report enabled to plan the implant in a more palatal position, resulting in a thicker BBT.

In the surgical phase, static computer-assisted immediate implant surgery with the pilot-drill surgical template resulted in correct three-dimensional implant placement. It must be acknowledged that a fully guided surgical technique can offer more accuracy and that a certain degree of deviation from the digital planning should be taken into consideration when using a pilot-drill surgical template [21, 22]. In the present case report, the temporary shell restorations were connected to the temporary abutment after immediate implant placement. This compensated for any deviations while still resulting in temporary restorations with screw-access holes on the palatal side in all cases, indicating that this method for immediate single-implant provisionalization can be used when a fully guided technique is not feasible.

In the restorative phase, the temporary restorations could be delivered in the same appointment as the implant placement. Whereas in the studies with a conventional workflow mentioned earlier [6, 7], impression taking after the surgery was necessary to design and manufacture a customized temporary restoration afterwards in the dental laboratory, as well as an extra appointment for the temporary restoration placement. Thus, chair time was reduced by using the digital workflow. Other advantages of a digital workflow for implant restoration procedures are that intraoral scanning lowers the procedure time and patient discomfort compared to conventional impression taking [23] and that CAD and computer-aided manufacturing (CAM) can lower the production time and fabrication costs of the restorations [24]. These are all possible arguments for choosing a digital workflow over a conventional workflow.

A downside of the temporary shell restorations used in these cases is the grayish discoloration of the metal temporary abutment through the composite and the PMMA. Due to this discoloration, the mean WES for the temporary restorations was just above the threshold of clinical acceptance (6.3 ± 0.6 on a scale of 0-10) [18]. However, this was not reflected by the patient-reported satisfaction for the provisional restorations (8.3 ± 0.6 on a scale of 1-10). It must be mentioned that the satisfaction score of the patient that started the treatment without a clinical crown might be biased. But aside from that, it can be assumed that patients are easily satisfied with a fixed temporary restoration, even when the color does not match the adjacent teeth. The fact that patients are more satisfied with the treatment outcome than the professional observers has been recorded before [7, 25], thus, further underlining the importance of including both parameters when evaluating esthetic outcomes.

Although the initial results of immediate implant placement and chairside provisionalization using a digital workflow are successful, the number of cases is limited. Clinical studies with a larger sample size are required to further evaluate the treatment outcomes. In conclusion, the three reported cases demonstrate the potential for predictable immediate implant placement and chairside provisionalization using a digital workflow.

## Figures and Tables

**Table 1:** Patient information.

	Case 1	Case 2	Case 3
Sex	Male	Male	Male
Age in years	22	63	29
Reason for treatment	Root fracture	Endodontic failure	Root fracture
Implant position	Upper left central incisor	Upper right central incisor	Upper left central incisor
Implant diameter	4.3 mm	4.3 mm	4.3 mm
Implant length	15 mm	15 mm	18 mm

**Table 2:** Timeline of the immediate implant placement, provisionalization, and final restoration treatment.

Treatment phase	Procedures
Diagnostic	Intraoral scans Cone beam computed tomography scan
Planning	Digital set-up Digital prosthetically driven implant planning Computer-aided design and milling of the temporary shell restoration Manufacturing of the surgical template
Surgical	Tooth extraction Buccal socket grafting Flapless static computer-assisted immediate implant surgery
Restorative	Chairside provisionalization
Restorative	Intraoral scans with a scanbody Computer-aided design and milling of the final abutment Porcelain veneering of the final restoration Placement of the final restoration

**Table 3:** Modified Plaque Index, modified Sulcus Bleeding Index, and Gingival Index (scores 0-3), keratinized mucosa width, Papilla Index (scores 0-4), and pocket probing depth at T12. Midbuccal mucosal level changes from Tpre to T12. Modified Pink Esthetic Score and White Esthetic Score (scores 0-10). Marginal bone level changes from T1 to T12. Buccal bone thickness at T12. Patient-reported satisfaction (scores 1-10).

	Case 1	Case 2	Case 3	Mean (SD)
Modified Plaque Index	Score 0	Score 0	Score 0	0.0 ± 0.0
Modified Sulcus Bleeding Index	Score 0	Score 1	Score 0	0.3 ± 0.6
Gingival Index	Score 0	Score 0	Score 0	0.0 ± 0.0
Keratinized mucosa width (mm)	≥2	≥2	≥2	≥2
Papilla Index				
Tpre mesial	Score 3	Score 1	Score 3	2.3 ± 1.2
Tpre distal	Score 3	Score 0	Score 2	1.7 ± 1.5
T12 mesial	Score 3	Score 1	Score 3	2.3 ± 1.2
T12 distal	Score 3	Score 0	Score 2	1.7 ± 1.5
Pocket probing depth (mm)	5	3	5	4.3 ± 1.2
Mid-buccal mucosal level changes (mm)^∗^	-0.51	-0.56	1.45	0.13 ± 1.5
Pink Esthetic Score				
Tpre	8	6	8	7.3 ± 1.2
Ttemp	8	6	8	7.3 ± 1.2
T12	9	7	9	8.3 ± 1.2
White Esthetic Score				
Tpre	4	7	N/A	5.5 ± 2.1
Ttemp	6	7	6	6.3 ± 0.6
T12	8	9	9	8.7 ± 0.6
Marginal bone level changes (mm)^∗^	0.06	0.02	0.11	0.06 ± 0.05
Buccal bone thickness (mm)				
M0 (at neck)	2.34	1.84	3.14	2.44 ± 0.66
M1	2.46	1.86	3.36	2.56 ± 0.76
M2	2.98	2.18	3.48	2.88 ± 0.66
M3	2.95	1.85	3.35	2.71 ± 0.78
M4	2.75	1.65	3.25	2.55 ± 0.82
M5	2.55	1.25	3.25	2.35 ± 1.02
Patient-reported satisfaction				
Tpre	5	5	5	5.0 ± 0.0
Ttemp	8	9	8	8.3 ± 0.6
T12	8	10	9	9.0 ± 1.0

Tpre: prior to the treatment; Ttemp: 6 weeks after temporary restoration placement; T1: 1 month after final restoration placement; T12: 1 year after final restoration placement; N/A: not applicable. ^∗^A positive value indicates tissue/bone level gain, and a negative value indicates loss.

## Data Availability

The authors confirm that the data supporting the findings of this study are available within the article.
